# Differential Effects of Alarmins on Human and Mouse Basophils

**DOI:** 10.3389/fimmu.2022.894163

**Published:** 2022-05-26

**Authors:** Adriana R. Gambardella, Remo Poto, Valentina Tirelli, John T. Schroeder, Gianni Marone, Fabrizio Mattei, Gilda Varricchi, Giovanna Schiavoni

**Affiliations:** ^1^ Department of Oncology and Molecular Medicine, Istituto Superiore di Sanità (ISS), Rome, Italy; ^2^ Department of Translational Medical Sciences, University of Naples Federico II, Naples, Italy; ^3^ Center for Basic and Clinical Immunology Research (CISI), University of Naples Federico II, Naples, Italy; ^4^ World Allergy Organization (WAO) Center of Excellence, Naples, Italy; ^5^ Core Facilities, Istituto Superiore di Sanità (ISS), Rome, Italy; ^6^ Division of Allergy and Clinical Immunology, Department of Medicine, Johns Hopkins University School of Medicine, Baltimore, MD, United States; ^7^ Institute of Experimental Endocrinology and Oncology (IEOS), National Research Council, Naples, Italy

**Keywords:** allergy, asthma, basophils, IL-4, IL-13, IL-25, IL-33, TSLP

## Abstract

Epithelial-derived alarmins (IL-33, TSLP, and IL-25) play an upstream role in the pathogenesis of asthma. Basophil-derived cytokines are a pivotal component of allergic inflammation. We evaluated the *in vitro* effects of IL-33, TSLP, and IL-25, alone and in combination with IL-3 on purified peripheral blood human basophils (hBaso) and bone marrow-derived mouse basophils (mBaso) in modulating the production of IL-4, IL-13, CXCL8 or the mouse CXCL8 equivalents CXCL1 and CXCL2. IL-3 and IL-33, but not TSLP and IL-25, concentration-dependently induced IL-4, IL-13, and CXCL8 release from hBaso. IL-3 synergistically potentiated the release of cytokines induced by IL-33 from hBaso. In mBaso, IL-3 and IL-33 rapidly induced IL-4 and IL-13 mRNA expression and protein release. IL-33, but not IL-3, induced CXCL2 and CXCL1 from mBaso. Differently from hBaso, TSLP induced IL-4, IL-13, CXCL1 and CXCL2 mRNA expression and protein release from mBaso. IL-25 had no effect on IL-4, IL-13, and CXCL1/CXCL2 mRNA expression and protein release even in the presence of IL-3. No synergism was observed between IL-3 and either IL-25 or TSLP. IL-3 inhibited both TSLP- and IL-33-induced CXCL1 and CXCL2 release from mBaso. Our results highlight some similarities and marked differences between the effects of IL-3 and alarmins on the release of cytokines from human and mouse basophils.

## Introduction

The epithelium represents a pivotal component of the innate immune system, providing a physical and immune barrier that is a first line of defense against environmental insults (1, 2). Damaged epithelial cells release a range of proinflammatory mediators causing barrier dysfunction and tissue remodeling (3, 4). Allergens, cytokines, microbial products, cigarette smoke extracts and physical stimuli induce the release of epithelium-derived alarmins including interleukin (IL)-33, thymic stromal lymphopoietin (TSLP) and IL-25 (3–7), also known as IL-17E (8). Although these alarmins possess some common characteristics, they markedly differ in immunological and biochemical characteristics (9–11). IL-33, an IL-1 superfamily member cytokine (9, 12), functions *via* IL-1RL1 (ST2)-mediated activation of several immune cells (12–14). TSLP is an IL-2 family member that activates the heterodimeric receptor TSLPR : IL-7Rα on a wide range of immune and non-immune cells (10, 15). IL-25/IL-17E is an unusual member of the IL-17 family of cytokines that binds, as a dimer, to the heterodimeric receptor IL-17RA : IL-17RB present on some immune cells (8). The upstream role of these epithelial alarmins has identified TSLP (16–18) and IL-33 (19, 20) as potential therapeutic targets for the treatment of T2-high and presumably also for T2-low asthma (20–22).

Although basophils account for 0.5% - 1% of all leukocytes in bone marrow and peripheral blood (23, 24), these cells play critical roles in clearing pathogens (25, 26), and initiating inflammatory processes in allergy (27–30), autoimmunity (31–34), immunodeficiencies (35, 36), and cancer (37–40). Most of these studies have been conducted in humans using peripheral blood basophils (hBaso) and often using mouse bone marrow-derived basophils (mBaso) as a model system. Since the beginning of these studies, there has been a debate regarding whether mouse basophils were indeed similar to human basophils (41, 42). It is now becoming evident that hBaso differ from mBaso with respect to cytokine and chemokine produced (43–46), surface receptors (47–49), responsiveness to immunological (46, 47, 49) and pharmacological stimuli (50). For instance, activated hBaso release a specific profile of cytokines (e.g., IL-3, IL-4, IL-13, CXCL8, VEGF-A) (36, 51–58), whereas mBaso express a wider spectrum of cytokines/chemokines (e.g., IL-3, IL-4, IL-6, IL-13, TNF-α, CXCL1, CXCL2, CXCL4) (43–45, 59, 60). Moreover, there has been controversy regarding the functional expression of TSLP receptor on human versus mouse basophils (46, 47, 49, 61). Finally, human and mouse basophils release IL-3 (58, 62). Therefore, there is the possibility of an autocrine priming mediated by IL-3 of basophils for functional response to alarmins.

Taken together, much remains to be learned about immunological differences between hBaso and mBaso. Accordingly, in this study we explored the *in vitro* effects of IL-33, TSLP, and IL-25, alone and in combination with IL-3 on the release of cytokines from highly purified human peripheral blood basophils and bone marrow-derived mouse basophils.

## Materials and Methods

### Reagents and Buffers

Bovine serum albumin (BSA), human serum albumin (HSA), [piperazine-N,N’ -bis (2-ethanesulfonic acid) (Pipes)] (Sigma-Aldrich, Milan, Italy), L-glutamine, antibiotic-antimycotic solution (10,000 IU penicillin, 10 mg/mL streptomycin, and 25 µg/mL amphotericin B), fetal calf serum (FCS), Iscove modified Dulbecco medium (IMDM) and Hanks’ balanced salt solution (GIBCO, Grand Island, NY, USA), RPMI 1640 with 25 mM HEPES buffer, Eagle’s minimum essential medium (Flow Laboratories, Irvine, UK), Percoll (Pharmacia Fine Chemicals, Uppsala, Sweden), rhIL-3, rhIL-33, rhIL-25, and rhTSLP (R& D System, Minneapolis, MN, USA), CD117 MicroBead (Miltenyi Biotech, Bologna, Italy), and HClO_4_ (Baker Chemical Co., Deventer, Netherlands) were commercially purchased. The Pipes (P) buffer was a mixture of 25 mM Pipes, 110 mM NaCl, 5 mM KCl, pH 7.37, referred to as P. P2CG, contains, in addition to P, 2 mM CaCl_2_ and 1 g/L dextrose (63); pH was titrated to 7.4 with sodium bicarbonate.

### Purification and Stimulation of Human Basophils

The study involving the use of human blood cells was approved by the Ethics Committee of the University of Naples Federico II (198/18), and written informed consent was obtained from all subjects involved in the study according to the recommendations from the Declaration of Helsinki. Basophils were purified from peripheral blood of healthy volunteers, aged 18-42 years, undergoing hemapheresis within the Immunohematology Unit at the University of Naples Federico II. Buffy coats were subjected to double-Percoll density centrifugation, which produced basophil-depleted cell and basophil-enriched cell suspensions (46). Basophils were purified from the basophil-enriched cell suspensions using the Basophil Isolation Kit II (Miltenyi, Biotec, Bologna, Italy). Basophils, with purity ~ 99% assessed by Alcian blue staining (64), were incubated at 37°C for 18 hours (52) in IMDM in the presence of increasing concentrations (1, 10, or 100 ng/ml) of the following cytokines: rhIL-3, rhIL-33, rhIL-25, and rhTSLP (R&D, Minneapolis, MN, USA). In some experiments, hBaso were stimulated with a combination of rhIL-3 (10 ng/ml) plus either rhIL-33 (10 ng/ml), rhTSLP (10 ng/ml) or rhIL-25 (10 ng/ml). At the end of these incubations, the cell-free supernatants were harvested and stored at -20°C for subsequent assay of IL-4, IL-13 and CXCL8 by ELISA.

### Differentiation and Purification of Bone Marrow-Derived Mouse Basophils

C57Bl/6 female mice were purchased from Charles River Laboratories (Calco, Italy) and housed in the animal facility at Istituto Superiore di Sanità (Rome, Italy), according to the current Italian law guidelines (D.Lgs.vo 26/14: Authorization no. 243/2016-PR, Prot. D9997.13). Bone marrow (BM) was collected from tibiae and femurs of 6-10 week old female C57BL/6 mice and cells were disaggregated by gently pipetting with RPMI 1640 culture medium (EuroClone, Pero, Milan, Italy) containing 10% FCS, 1% penicillin, 1% fungizone, 1% streptomycin. To eliminate the erythrocytes, the BM cell suspension was incubated with 4 ml of ammonium-chloride-potassium (ACK) lysis buffer for 4 minutes at 22°C. BM cells (30 x 10^6^) were cultured in complete RPMI 1640 supplemented with 1% of sodium pyruvate (Basophil medium) plus 2 ng/ml of recombinant mouse IL-3 (rmIL-3; R&D Systems, Minneapolis, MN, USA). At days 4 and 7 of differentiation, non-adherent cells were harvested, centrifuged (5 minutes at 400 x g, 4°C) and re-seeded in a new flask containing fresh Basophil medium and 2 ng/ml of rmIL-3. At day 10, cells were harvested and stained with the following monoclonal antibodies (mAbs) for flow cytometry analyses: PE anti-mouse CD49b (clone DX5, Miltenyi Biotec), FITC anti-mouse FcεR1 (clone MAR-1), PE-Cy7 anti-mouse CD117/c-kit (clone 2B8), APC anti-mouse CD11c (clone N418) or APC anti-mouse CD200R (clone Ba13) (all from Biolegend, San Diego, CA, USA). Mouse basophils (FcεR1^+^ CD11c^-^ c-kit^-^) were purified by fluorescence activated cell sorting (FACS) using a MoFlo Astrios EQ cell sorter (Beckman Coulter, Brea, CA, USA). The obtained mBaso population was routinely > 98% pure (see **Figure S1**).

### Stimulation of Mouse Basophils

Sorted mBaso were incubated (10^6^ cells/ml) for various times at 37°C with graded concentrations (1, 10, or 100 ng/ml) of the following cytokines: rmIL-3, rmIL-33 (BioLegend, San Diego, CA, USA), rmIL-25 and rmTSLP (R&D Systems, Minneapolis, MN, USA). In some experiments, mBaso were stimulated with a combination of rmIL-3 (1 ng/ml) plus either rmIL-33 (1 or 10 ng/ml), rmTSLP (1 or 10 ng/ml) or rmIL-25 (1 or 10 ng/ml). Cells and culture supernatants were harvested at 4 h and 24 h for further analyses.

### Gene Expression Analysis With qRT-PCR

Total RNA was extracted from mBaso by using TRIsure reagent (Bioline, London, UK). Messenger RNA was reverse transcribed by means of Tetro cDNA Synthesis Kit (Bioline). Quantitative reverse transcription-PCR (qPCR) was performed using Sensimix Plus SYBR Kit containing the fluorescent dye SYBR Green (Bioline) and by means of an ABI 7500 Real-time PCR system (Applied Biosystems, Thermo Fisher Scientific, Waltham, MA, USA). Forward and reverse primers (**Table SI**) were purchased from Eurofin Genomics (Ebersberg, Germany). Triplicates were performed for each experimental point and data were normalized to HPRT (2-ΔCt method).

### ELISA Assays

Human IL-4, IL-13 and CXCL8 were assessed in duplicate samples using ELISA kits according to the manufacturer’s instructions (Quantikine ELISA Kit) (R&D Systems, Minneapolis, MN, USA). The ELISA detection range was 31-2,000 pg/ml (IL-4), 125-4,000 pg/ml (IL-13) and 31-2,000 pg/ml (CXCL8). mBaso were cultured in 48 multiwells at 10^6^ cells/ml with single or combined cytokines, as described above. Supernatants were collected after 24 hours to perform ELISA assays by using specific kits to evaluate the production of murine IL-4 (Biolegend), IL-13 (Thermo Fisher Scientific), CXCL1 and CXCL2 (R&D Systems, Minneapolis, MN, USA). The ELISA detection range was 2-125 pg/ml (mIL-4), 7.8-500 pg/ml (mIL-13) 15.6- 1,000 pg/ml (mCXCL1), 7.8- 500 pg/ml (mCXCL2).

### Flow Cytometry of IL-33, IL-25 and TSLP Receptors

mBaso were cultured for 24 hours alone (untreated) or in the presence of IL-3 at graded concentrations (1, 10 or 100 ng/ml). Cells were then stained with PE-Cy7 anti-mouse ST2 (clone RMST2-2; Thermo Fisher Scientific), PE anti-mouse TSLPR (clone 22H9; BD Biosciences, Franklin Lakes, NJ) and APC anti-mouse IL-25R/IL-17RB (clone 9B19) (Biolegend, San Diego, CA, USA) and analyzed by flow cytometry.

### Statistical Analysis

All data were analyzed for statistical significance by means of Prism 8 (GraphPad software). Values from groups were compared by Student’s *t*-test based on the parametric or nonparametric distribution of the continuous variables. One-way ANOVA analysis of variance was performed to compare means among multiple groups, followed by *post hoc* testing (Tukey test). Values were considered significant when the probability was below the 5% confidence level (*p* < 0.05).

## Results

### Effects of IL-3 and Alarmins on Cytokine Release from Human Basophils

**Figure 1:**
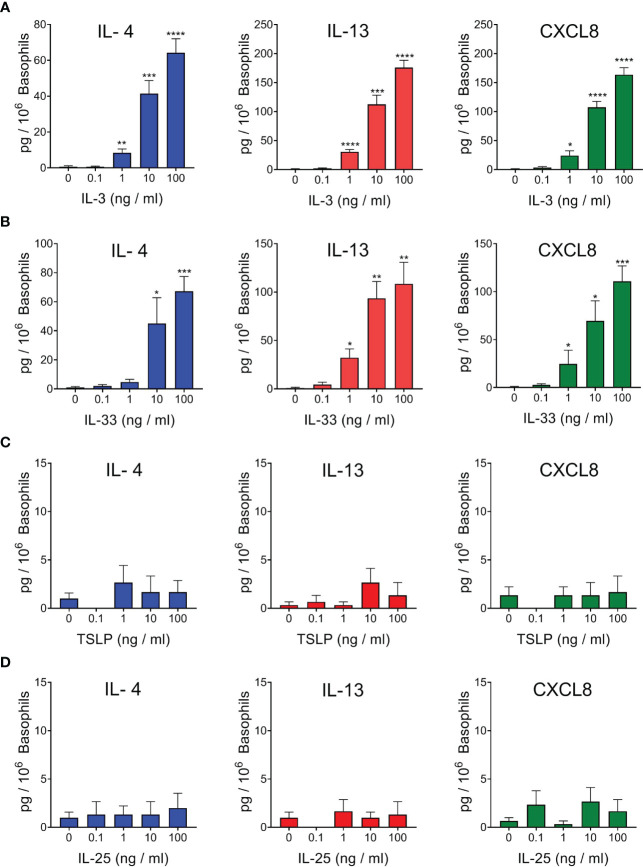
Effects of increasing concentrations of rhIL-3 **(A)**, rhIL-33 **(B)**, rhTSLP **(C)**, and rhIL-25 **(D)** on IL-4, IL-13, and CXCL8 release from different preparations of human basophils (n=5). Basophils were incubated (18 hours at 37°C) with the indicated concentrations of cytokines. At the end of incubations, cell-free supernatants were collected and analyzed for IL-4, IL-13, and CXCL8 protein by ELISA. *p < 0.05; **p < 0.01; ***p < 0.001; ****p < 0.0001.

In a first series of experiments we evaluated the effects of incubation (20 h at 37°C) with IL-3 on purified basophils from healthy donors (hBaso). **Figure 1A** shows that IL-3 (1 to 100 ng/ml) concentration-dependently induced the secretion of IL-13 and CXCL8 and, to a lesser extent, of IL-4 from five different preparations of hBaso. We also investigated the effects of alarmins (IL-33, TSLP, and IL-25) on the production of cytokines from hBaso. **Figure 1B** indicates that IL-33 (1 to 100 ng/ml) caused a concentration-dependent release of IL-4, IL-13, and CXCL8.

The heterodimeric receptor for TSLP, composed of TSLPR and IL-7Rα, has not been found on basophils from healthy donors and allergic subjects (47) and the activating property of TSLP on human basophils has been apparently conflicting (46, 47, 49, 61, 65). In all our four independent experiments, we found that TSLP (0.1 to 100 ng/ml) did not induce the release of detectable levels of IL-4, IL-13, and CXCL8 from purified basophils obtained from healthy donors (**Figure 1C**). IL-25 (IL-17E) is an alarmin which belongs to the IL-17 family and activates the heterodimeric receptor IL-17RA : IL-17RB (8). **Figure 1D** shows the results of four independent experiments, indicating that IL-25 (0.1 to 100 ng/ml) did not cause the release of IL-4, IL-13, and CXCL8 from hBaso.

### Effects of IL-3 on Alarmin-Induced Cytokine Release from Human Basophils

**Figure 2:**
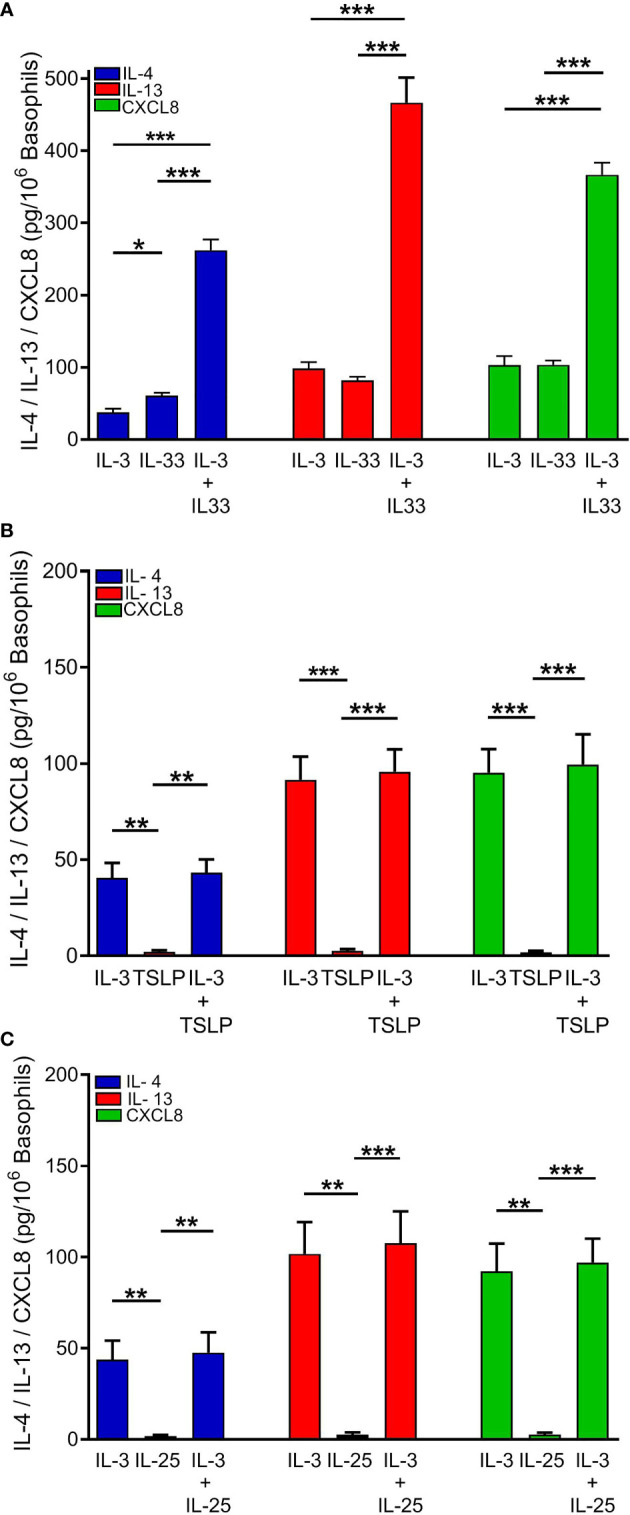
Effects of rhIL-3 (10 ng/ml), alone and in combination with **(A)** rhIL-33 (10 ng/ml), **(B)** rhTSLP (10 ng/ml), or **(C)** rhIL-25 (10 ng/ml), on IL-4, IL-13, and CXCL8 released from different preparations of human basophils (n = 4). Basophils were incubated (18 hours at 37°C) with the indicated concentrations of cytokines. At the end of incubations, cell-free supernatants were collected and analyzed for IL-4, IL-13, and CXCL8 protein by ELISA. *p < 0.05; **p < 0.01; ***p < 0.001.

Human basophils express the heterodimeric IL-3 receptor composed of the IL-3Rα (CD123) and the βc chain (61, 66). IL-3 is best known for its ability to mediate priming effects when combined with a variety of different co-stimuli (58, 67–70). As also shown in **Figure 1**, IL-33 (10 ng/ml) and IL-3 (10 ng/ml) caused IL-4 release from hBaso. Incubation of hBaso with IL-3 (10 ng/ml) and IL-33 (10 ng/ml) synergistically potentiated IL-4 release from hBaso (*p* < 0.001). IL-3 and IL-33 induced the secretion of both IL-13 and CXCL8 from hBaso. **Figure 2A** also shows that incubation of hBaso with IL-3 synergistically potentiated the capacity of IL-33 to induce IL-13 (*p* < 0.001) and CXCL8 (*p* < 0.001).

Similar experiments were performed to investigate the possible interactions between IL-3 and TSLP or IL-25 on the release of cytokines from hBaso. **Figure 2B** shows the results of four independent experiments performed with different preparations of hBaso, indicating that the lack of activating effect of TSLP on the release of cytokines (IL-4, IL-13, and CXCL8) was not modified by co-incubation with IL-3 (10 ng/ml). Similarly, hBaso were essentially unresponsive to IL-25 even when cells were co-incubated with IL-3 (10 ng/ml) (**Figure 2C**).

### Effects of IL-3 and Alarmins on Cytokine Release from Mouse Basophils

**Figure 3:**
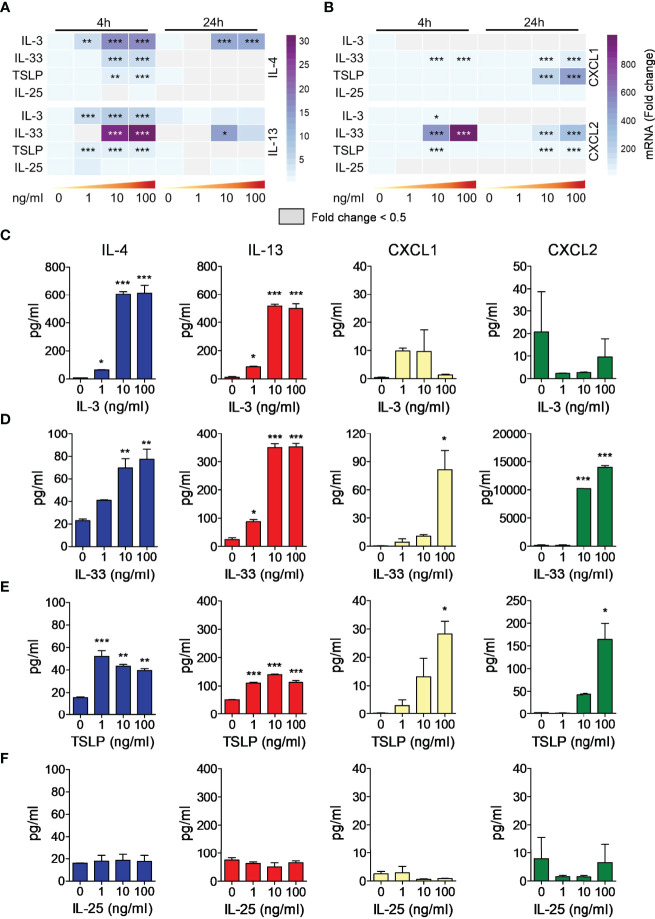
Effects of rmIL-3, rmIL-33, rmTSLP, or rmIL-25 on cytokine (IL-4, IL-13) and chemokine (CXCL1, CXCL2) mRNA expression and protein release by mBaso. Purified mBaso (10^6^ cells/ml) were stimulated with the indicated concentrations of rmIL-3, rmIL-33, rmTSLP, or rmIL-25. Expression of IL-4 and IL-13 **(A)**, CXCL1 and CXCL2 **(B)** mRNAs after 4 and 24 hours of stimulation was assessed by qRT-PCR. Data in heatmap represent the mean fold increase of mRNA normalized to HPRT and with respect to untreated (NT) controls at 4 h +/- SD. Concentrations of IL-4, IL-13, CXCL1 and CXCL2 in culture supernatants of mBaso incubated (24 hours at 37°C) with the indicated concentrations of rmIL-3 **(C)**, rmIL-33 **(D)**, rmTSLP **(E)**, or rmIL-25 **(F)** were measured by ELISA. Data represent the mean amount of released cytokines +/- SD in culture triplicates. One representative experiment out of at least three replicates is shown. *p < 0.05; **p < 0.01; ***p < 0.001.

To evaluate the effects of IL-3 and alarmins on cytokine release by mBaso we employed highly purified BM-derived basophils. After 10 days of differentiation in the presence of IL-3 (71), basophils were FACS-sorted as FcεR1^+^ CD11c^-^ kit^-^ to remove contaminating mast cells (FcεR1^+^, c-kit^+^) and dendritic cells (CD11c^+^), yielding more than 95% pure population (**Figure S1**). Sorted mBaso were cultured in the presence of graded concentrations of IL-3, IL-33, TSLP or IL-25. Cytokine and chemokine expression was evaluated after 4 h and 24 h, while the protein release in the culture supernatants was measured after 24 h of incubation. IL-3 induced both mRNA expression (**Figures 3A** and **S2A** for statistical analysis) and protein release (**Figure 3C**) of IL-4 and IL-13 in similar amounts and a concentration-dependent manner, with maximum efficacy at 10 ng/ml. Stimulation of mBaso with IL-33 also induced concentration-dependent expression (**Figures 3A** and **S2B**) and production (**Figure 3D**) of IL-4 and IL-13, the latter released in notably higher amounts (~ 5-fold) with respect to IL-4 (**Figure 3D**). Of note, TSLP induced low but significant increases in the expression of IL-4 and IL-13 mRNA (**Figures 3A** and **S2C**) and secretion of protein (**Figure 3E**) levels in mBaso stimulated with just 1 ng/ml. In contrast, IL-25 failed to induce any of these cytokines by mBaso at all concentrations tested (**Figures 3A, F** and **S2D**). We also measured the expression and release of the chemokines CXCL1 and CXCL2, two mouse functional homologues of human CXCL8 (72). IL-3 did not increase the expression of CXCL1 and CXCL2 in mBaso (**Figures 3B** and **S2A**). Both IL-33 and TSLP could induce these two chemokines in mBaso. In fact, TSLP dose-dependently enhanced the expression (**Figures 3B** and **S2C**) and production (**Figure 3E**) of both chemokines, while IL-33 showed a greater capacity to induce CXCL2, compared to CXCL1, both at mRNA (**Figures 3B** and **S2B**) and protein level (**Figure 3D**). In contrast, stimulation with IL-25 failed to induce either CXCL1 or CXCL2 expression (**Figures 3B** and **S2D**) and protein release (**Figure 3F**) in mBaso.

### Effects of IL-3 on Alarmin-Induced Cytokine Release from Mouse Basophils

**Figure 4:**
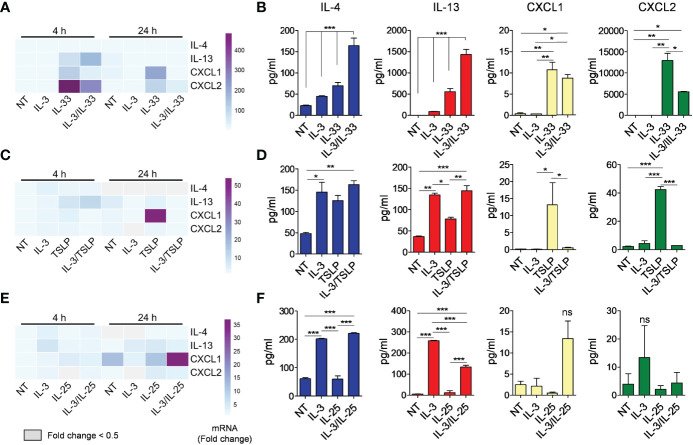
Effects of rmIL-3 alone and in combination with rmIL-33, rmTSLP, or rmIL-25 on cytokine (IL-4, IL-13) and chemokine (CXCL1, CXCL2) mRNA expression and release from mBaso. Purified mBaso (10^6^ cells/ml) were stimulated with rmIL-3 (1 ng/ml) alone and in combination with **(A, B)** rmIL-33 (10 ng/ml), **(C, D)** rmTSLP (10 ng/ml) or **(E, F)** rmIL-25 (10 ng/ml). **(A, C, E)** mRNA expression was assessed by qRT-PCR after 4 and 24 hours of stimulation. Data in heatmap represent the mean fold increase of mRNA normalized to HPRT and with respect to untreated (NT) controls at 4 h +/- SD. **(B, D, F)** Concentrations of IL-4, IL-13, CXCL1 and CXCL2 in culture supernatants of mBaso incubated (24 h at 37°C) with the indicated combinations were measured by ELISA. NT: not stimulated. Data represent the mean amount of released cytokines +/- SD in culture triplicates. One representative experiment out of at least two replicates is shown. *p < 0.05; **p < 0.01; ***p < 0.001 ns, Not Significant.

Next, we analyzed whether IL-3 could synergistically increase the response of mBaso to alarmin stimulation. To this end, we cultured mBaso with a suboptimal dose of IL-3 (1 ng/ml) in combination with an optimal dose of IL-33 (10 ng/ml), TSLP (10 ng/ml) or IL-25 (10 ng/ml). IL-3 strongly synergized with IL-33 in inducing IL-4 and IL-13 mRNA expression (**Figures 4A** and **S3A** for statistical analysis) and protein secretion (**Figure 4B**) by mBaso. In contrast, IL-3 decreased the expression (**Figures 4A** and **S3A** for statistical analysis) and production (**Figure 4B**) of CXCL1 and CXCL2 induced by IL-33. Combination of IL-3 (1 ng/ml) with TSLP (10 ng/ml) did not enhance IL-4 and IL-13 production while it inhibited the expression (**Figures 4C** and **S3B** for statistical analysis) and release (**Figure 4D**) of CXCL1 and CXCL2 with respect to TSLP alone. IL-3 (1 ng/ml) failed to prime mBaso to IL-25 (10 ng/ml) stimulation, with regard to cytokine and chemokine secretion (**Figure 4F**). Moreover, this combined treatment resulted in a slight but significant reduction in the levels of IL-13 secreted compared to those seen with IL-3 alone (**Figures 4E, F** and **S3C** for statistical analysis). Although we found a synergistic effect of IL-3 and IL-25 in inducing the mRNA expression of CXCL1 at 24 h (**Figures 4E** and **S3C**), the levels of released CXCL1 were not significantly higher compared to those from mBaso exposed to the single cytokines or left untreated (**Figure 4F**).

### Modulation of Alarmin Receptor Expression in mBaso by IL-3

**Figure 5:**
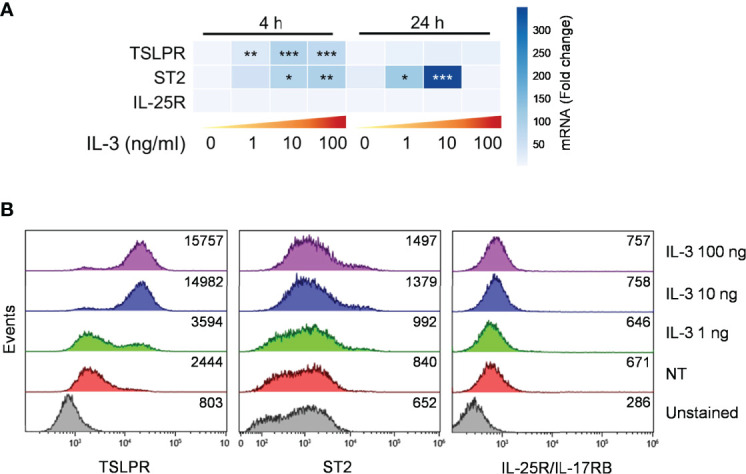
Expression of alarmin receptors by murine basophils. Sorted mBaso (10^6^ cells/ml) were cultured (24 hours at 37°C) alone (NT) or in the presence of the indicated concentrations of rmIL-3. Cells were then analyzed for expression of ST2, TSLPR and IL-25/IL-17RG by **(A)** qRT-PCR and **(B)** flow citometry. **(A)** Data in heatmap represent the mean fold mRNA increase normalized to HPRT and with respect to untreated (0) controls at 4 h +/- SD. **(B)** Numbers inside each plot represent mean fluorescence intensity values. One representative experiment out of two is shown. *p < 0.05; **p < 0.01; ***p < 0.001.

We asked whether the effects of IL-3 in modulating the response of mBaso to alarmins could be attributable to its differential capacity to modulate the expression of membrane receptors for these alarmins. Thus, we stimulated mBaso with graded concentrations of IL-3 and analyzed the expression of the specific subunits of each alarmin, namely ST2, TSLPR and IL-17RB, by qRT-PCR and flow cytometry. We observed a IL-3 (1-100 ng/ml) concentration-dependent increase in the mRNA (**Figure 5A**) and membrane protein (**Figure 5B**) of ST2 and TSLPR, but this did not affect the expression of IL-25R. Overall, these data indicate that IL-3 may enhance the responsiveness of mBaso to stimulation with IL-33 at least in part by increasing the expression of its specific receptor on basophil membranes.

## Discussion

Increasing evidence supports the concept that the epithelium constitutes a key component of the innate immune system, providing a physical and immune-modulatory barrier that is a first line of defense against environmental insults (1, 2). Alarmins (TSLP, IL-33, IL-25/IL-17E), constitutively expressed in epithelial cells, are the first reactors to immunologic, infectious, neoplastic and traumatic insults and rapidly initiate innate and adaptive immune responses (11, 14, 73). In this study, we have analyzed the *in vitro* effects of IL-33, TSLP, and IL-25, alone and in combination with IL-3, on the production of cytokines from highly purified peripheral blood human basophils and bone marrow-derived mouse basophils. Our results showed some similarities and several qualitative and quantitative differences between the effects of these alarmins on human and mouse basophils.

IL-3, at all concentrations tested, markedly induced IL-13 and CXCL8 secretion from hBaso, whereas it has less effect on IL-4, consistent with previous reports (46, 54, 74). By contrast, IL-3 concentration-dependently caused a rapid and long-lasting increase in both IL-4 and IL-13 mRNA expression and in the secretion of these cytokines from mBaso.

Another striking difference between human and mouse basophils was the response to TSLP. We confirmed the recent results by other investigators (46, 47) by showing that a wide spectrum of TSLP did not induce the release of cytokines IL-4 and IL-13 from hBaso. We also extended the previous findings by showing that TSLP did not cause CXCL8 release from hBaso. In contrast, TSLP caused IL-4, IL-13, CXCL1 and CXCL2 mRNA expression and the release of these cytokines from mBaso. The latter findings support a role of TSLP in the differentiation and activation of basophils, as previously demonstrated in different mouse models (37, 75–77).

The distinct effects of TSLP on cytokine release from hBaso and mBaso could be explained by different methods of cell preparations. hBaso purified from peripheral blood of normal donors are not exposed to IL-3, while mBaso are obtained from bone marrow-derived precursors differentiated in the presence of IL-3. For the above reasons, we recently examined the effects of TSLP, alone and in presence of IL-3, on culture-derived human basophils (CDBA) (61). CDBA were obtained by culturing CD34^+^ precursor cells in the presence of IL-3. In these experiments, IgE-sensitized and non IgE-sensitized basophils stimulated with TSLP alone or in combination with IL-3 did not release IL-4 and IL-13 (61).

The activating effects of IL-33 on hBaso and mBaso were qualitatively similar but presented some quantitative differences. This alarmin induced the secretion of similar amounts of IL-4 and IL-13 from hBaso but higher levels of IL-13 compared to IL-4 in mBaso. CXCL8 in hBaso was induced by IL-33 at similar levels as CXCL1 in mBaso. In mBaso, IL-33 induced massive amounts of CXCL2. Accordingly, a recent report has shown that IL-33 induces similar amounts of mRNA and protein expression of the neutrophil-attracting mediators CXCL1 and CXCL2 by bone marrow-derived basophils in a mouse model of skin inflammation (60).

IL-25/IL-17E is a member of the IL-17 cytokine family and is known to activate a limited number of immune cells (11). As previously reported (46), a wide range of concentrations of IL-25 did not induce the release of IL-4 and IL-13 from hBaso. Our results extend on these findings in showing that IL-25 displayed no capacity to induce CXCL8 secretion from basophils purified from unselected healthy volunteers. The current and previous results (46) indicate that IL-25 does not induce the release of several cytokines from basophils purified from peripheral blood of healthy volunteers. IL-25 also failed to induce IL-4, IL-13, CXCL1 and CXCL2 mRNA expression in mBaso or to stimulate the production of these cytokines. Notably, we observed that IL-25 even inhibits the release of IL-13 induced by IL-3 from mBaso. It should be noted that in different experimental models it has been reported that IL-25 inhibits the activating property of IL-22 (78) and of Poly I:C (79).

IL-3 is well known for its ability to synergize with a wide range of immunological stimuli to augment the release of several mediators from human basophils (51, 54, 58, 74, 80). Consistent with the results previously published (57, 58, 80), we found that exposure of hBaso to IL-3 synergistically potentiated the activating effects of IL-33 for the production of IL-13, IL-4, and CXCL8 from these cells. The synergistic interaction between IL-3 and IL-33 was more marked with respect to IL-13 secretion compared to IL-4 release from hBaso. These findings are explained, in part, by evidence that IL-3 significantly induces the expression of ST2 – the IL-33 receptor on human basophils (80, 81). These findings might have translational relevance in various immune disorders. Human and mouse basophils release IL-3 (58, 62). Therefore, the synergistic interaction between IL-3 and certain alarmins on the release of immunomodulatory cytokines from hBaso and mBaso might be relevant in the pathophysiology of different basophil-mediated disorders such as allergic diseases (27–30), parasitic infections (82, 83), autoimmunity (31–34), and cancer (37–40).

To the best of our knowledge, it had never been demonstrated whether a similar priming effect of IL-3 is also exerted on mBaso activated by different stimuli. In this study we have examined the possible interactions between IL-3 and three alarmins on the production of cytokines from mBaso. Incubation of mBaso with low-dose (1 ng/ml) of IL-3 synergistically potentiated the releasing activity of IL-33 (10 ng/ml) on the secretion of both IL-4 and IL-13. The relevance of these findings is supported by the observation that IL-3 and IL-33 synergize *in vivo* to produce IL-4 from mBaso (84). Interestingly, the observation that IL-3 increased the expression of ST2 on mBaso suggests that IL-3 may amplify IL-33 responsiveness through up-regulation of ST2. On the other hand, the finding that IL-3 inhibited the production of CXCL1 and CXCL2 induced by IL-33 suggests the involvement of downstream modulation pathways.

It has been reported that IL-3 induces the expression of TSLPR but not of IL-7Rα on human basophils (47). In the latter study, basophils were unresponsive to TSLP. In keeping with this, IL-3 markedly upregulated the expression of TSLPR on mBaso, both mRNA and cell surface protein, but did not enhance their response to TSLP. Interestingly, incubation of mBaso with low-dose (1 ng/ml) IL-3 inhibited the production of CXCL1 and CXCL2 from mBaso stimulated by TSLP, as observed with IL-33. Incubation of hBaso with IL-3 did not modify the lack of response to IL-25 of these cells. Similar to hBaso, IL-25 had no effect on IL-3-stimulated mBaso with respect to IL-4/IL-13 mRNA expression and protein level and this correlated with failure by IL-3 to increase IL-17RB expression on mBaso.

Collectively, our results highlight different levels of complexity when examining the effects of alarmins (TSLP, IL-33, and IL-25) on the production of different cytokines from hBaso and mBaso. In particular, IL-33 appears to have comparable activating effects on both human and mouse basophils. By contrast, TSLP is a more selective activator of mBaso, but not of hBaso. Moreover, IL-25 seemingly does not activate mouse or human basophils. The above information appear of translational relevance for efforts at elucidating the complex immunological functions of alarmins in allergic disorders. It is commonly thought that epithelial-derived alarmins (IL-33, TSLP, IL-25) play an upstream role in the pathogenesis of asthma (2, 10, 15, 73) where basophil-derived cytokines are a pivotal component of allergic inflammation (26, 67, 85). Our results suggest that individual alarmins, which have different and specific roles in the activation of basophils, presumably play distinct roles in the pathogenesis of allergic disorders.

We also found quantitative differences in the activating properties of IL-3 and alarmins. For instance, the IL-3 and IL-3 plus IL-33 conditions markedly increased IL-13 more than IL-4 production from hBaso, but had similar effects on the release of these cytokines from mBaso. Another striking difference between human and mouse basophils was found when examining the synergism between IL-3 and alarmins. IL-3 synergistically potentiated the release of several cytokines induced by IL-33 in human and murine basophils. In contrast, combination of IL-3 with either IL-33 or TSLP caused an inhibitory effect in mBaso, but not in hBaso, in the production of neutrophil-attracting chemokines. Finally, IL-3 and IL-25 had no synergistic or additive effect in both human and murine basophils.

The prominent differences in the activating properties of IL-3 and of the three alarmins examined in this study are not surprising. IL-3, together with IL-5 and GM-CSF, belongs to the β common chain (βc) cytokine family (61). IL-33 is an IL-1 family member that activates the ST2 receptor enabling the recruitment of the co-receptor IL-1RAcP (86). TSLP is a member of the IL-2 family of helical cytokines, which binds to the heterodimeric TSLPR, namely the IL-7Rα receptor chains (10). IL-25/IL-17E is an unusual member of the IL-17 family of cytokines which binds as a disulphide-linked dimer to IL-17RA-IL-17RB receptor chains (11). Moreover, IL-3 and the three alarmins activate distinct signaling and transcription factors (11, 50, 87). Therefore, it is not surprising that these different cytokines produce distinct physiological effects on human and mouse basophils.

Bone marrow-derived murine basophils are often used as a model system for studies of the role of these cells in human health and disease (37, 45, 50, 59, 60, 62). Our results illustrate several differences in the release of some key cytokines induced by IL-3 and alarmins, alone and in combination, from hBaso and mBaso. Although mouse basophils represent useful models for understanding the role of these cells in different experimental disorders, our findings suggest caution when making generalizations to human disease.

Another important technical issue is the purity of cells with which one works. Basophils account for 0.5% - 1% of all leukocytes in bone marrow and peripheral blood (23, 24) and are difficult to purify to the homogeneity. Our experiments were carefully executed with highly purified human and mouse basophils in order to exclude the possibility that contaminating cells, in particular IgE^+^ dendritic cells (47, 88), may have influenced the expression and production of cytokines. However, we cannot exclude the possibility that during the purification procedures to obtain highly purified basophils we could have lost a small percentage of cells responsive to some of the examined alarmins (e.g., TSLP, IL-25).

Our study has some limitations that should be pointed out. First, mouse basophils were generated by culturing murine bone marrow cells for 14 days with IL-3. Murine basophils generated in this manner have an activated phenotype (82, 89) and differ from naïve primary mature basophils (50). Therefore, bone marrow-derived mBaso cannot be directly compared to mature hBaso obtained from peripheral blood. Another technical issue stems from the purification procedures to obtain highly purified human and mouse basophils. We used flow cytometry to purify mBaso and there is a possibility that this procedure might have activated murine basophils. Finally, Gonzales and collaborators have recently identified four phenotypically distinct subpopulations of human peripheral blood basophils (90) and Pellefigues and coworkers have provided some evidence of the existence of mBaso functional heterogeneity (50). In our study, we did not address the emerging issue of possible existence of human and mouse basophil heterogeneity. Another obvious question is if mBaso from other tissues are similar to bone marrow-derived mBaso. There is preliminary evidence that mBaso can acquire distinct characteristics depending on the different tissue locations (50). Collectively, these observations highlight the intrinsic complexity of analyzing and comparing the *in vitro* functions of human versus mouse basophils.

In conclusion, our study highlights some similarities and several differences in response to alarmins between hBaso and bone marrow-derived mBaso. The results of this study can thereby serve as a reference guide for scientists of basophil biology to interpret the results obtained with human basophils versus those of mouse. Such information appears of translational relevance for efforts aimed at elucidating the complex biological functions of both alarmins and basophils in health and disease.

## Data Availability Statement

The original contributions presented in the study are included in the article/**Supplementary Material**. Further inquiries can be directed to the corresponding authors.

## Ethics Statement

The study involving the use of human blood cells was approved by the Ethics Committee of the University of Naples Federico II (198/18), and written informed consent was obtained from all subjects involved in the study according to the recommendations from the Declaration of Helsinki. The patients/participants provided their written informed consent to participate in this study. C57Bl/6 female mice were housed in the animal facility at Istituto Superiore di Sanità (Rome, Italy), according to the current Italian law guidelines (D.Lgs.vo 26/14: Authorization no. 243/2016-PR, Prot. D9997.13).

## Author Contributions

ARG, RP, VT, JTS, GM, GV, GS designed and conducted laboratory studies. ARG, GM, FM, GV, GS designed and performed data analysis. ARG, RP, JTS, GM, FM, GV, GS interpreted data. ARG, RP, GM, FM, GV, GS drafted the manuscript and interpreted data. ARG, JTS, GM, GV, GS edited the manuscript. All the authors approved the manuscript.

## Funding

This work was supported in part by grants from the CISI-Lab Project (University of Naples Federico II), TIMING Project and Campania Bioscience (Regione Campania) to GM, GV, AIRC (IG 21366) to GS, and by grant R01 AI141486 [National Institute of Allergy and Infectious Diseases (NIAID), National Institutes of Health (NIH)] to JS.

## Conflict of Interest

The authors declare that the research was conducted in the absence of any commercial or financial relationships that could be construed as a potential conflict of interest.

## Publisher’s Note

All claims expressed in this article are solely those of the authors and do not necessarily represent those of their affiliated organizations, or those of the publisher, the editors and the reviewers. Any product that may be evaluated in this article, or claim that may be made by its manufacturer, is not guaranteed or endorsed by the publisher.

## Abbreviations

ACK, Ammonium-Chloride-Potassium; BM, Bone marrow; BSA, Bovine serum albumin; CDBA, culture-derived human basophils; FACS, Fluorescence-activated cell sorter; FCS, Fetal calf serum; hBaso, Human basophils; HSA, Human serum albumin; IL, Interleukin; IMDM, Iscove modified Dulbecco media; mBaso, Mouse basophils; OD, Optical density; qRT-PCR, Quantitative reverse transcription-PCR; rmIL, recombinant mouse interleukin; TSLP, Thymic stromal lymphopoietin; VEGF, Vascular endothelial growth factor.
