# Supplementary material for: Differential Effects of Alarmins on Human and Mouse Basophils
Source: Front Immunol. 2022 May 26;13:894163. doi: 10.3389/fimmu.2022.894163 (PMC9177950; doi:10.3389/fimmu.2022.894163)

Supplementary Materials

# Supplementary Table

**Table SI.** List of forward and reverse primers used for real-time quantitative PCR.

| **Gene** | **Forward and Reverse Primers (5’-3’)** | **NCBI Accession Number** |
| --- | --- | --- |
| **IL-4** | ACAGGAGAAGGGACGCCAT  GAAGCCCTACAGACGAGCTCA | [NM_021283.2](https://www.ncbi.nlm.nih.gov/entrez/viewer.fcgi?db=nucleotide&id=226874825) |
| **IL-13** | AGACCAGACTCCCCTGTGCA  TGGGTCCTGTAGATGGCATTG | NM_008355.3 |
| **ST2 (IL1RL1)** | GAATGGGACTTTGGGCTTTG  CAGGACGATTTACTGCCCTCC | [NM_001025602.3](http://www.ncbi.nlm.nih.gov/entrez/viewer.fcgi?db=nucleotide&id=659667962) |
| **CXCL1** | CACCCAAACCGAAGTCATAGC  TTACTTGGGGACACCTTTTAGC | NM_008176.3 |
| **CXCL2** | TCCAAAAGATACTGAACAAAGGC  CACATCAGGTACGATCCAGG | NM_009140.2 |
| **TSLPR** | GCTCCTTCCCTGGACTCTTT  GACAGGCACAGGATTTGTGA | NM_001310694.1 |
| **IL-17RB** | CGGGCGCCTGGATAAGAGGACCC  CATCCACTCTGGAGATGGCCCTGT | NM_019583.3 |
| **HPRT** | CTGGTGAAAAGGACCTCTCG  TGAAGTACTCATTATAGTCAAGGGCA | NM_013556.2 |


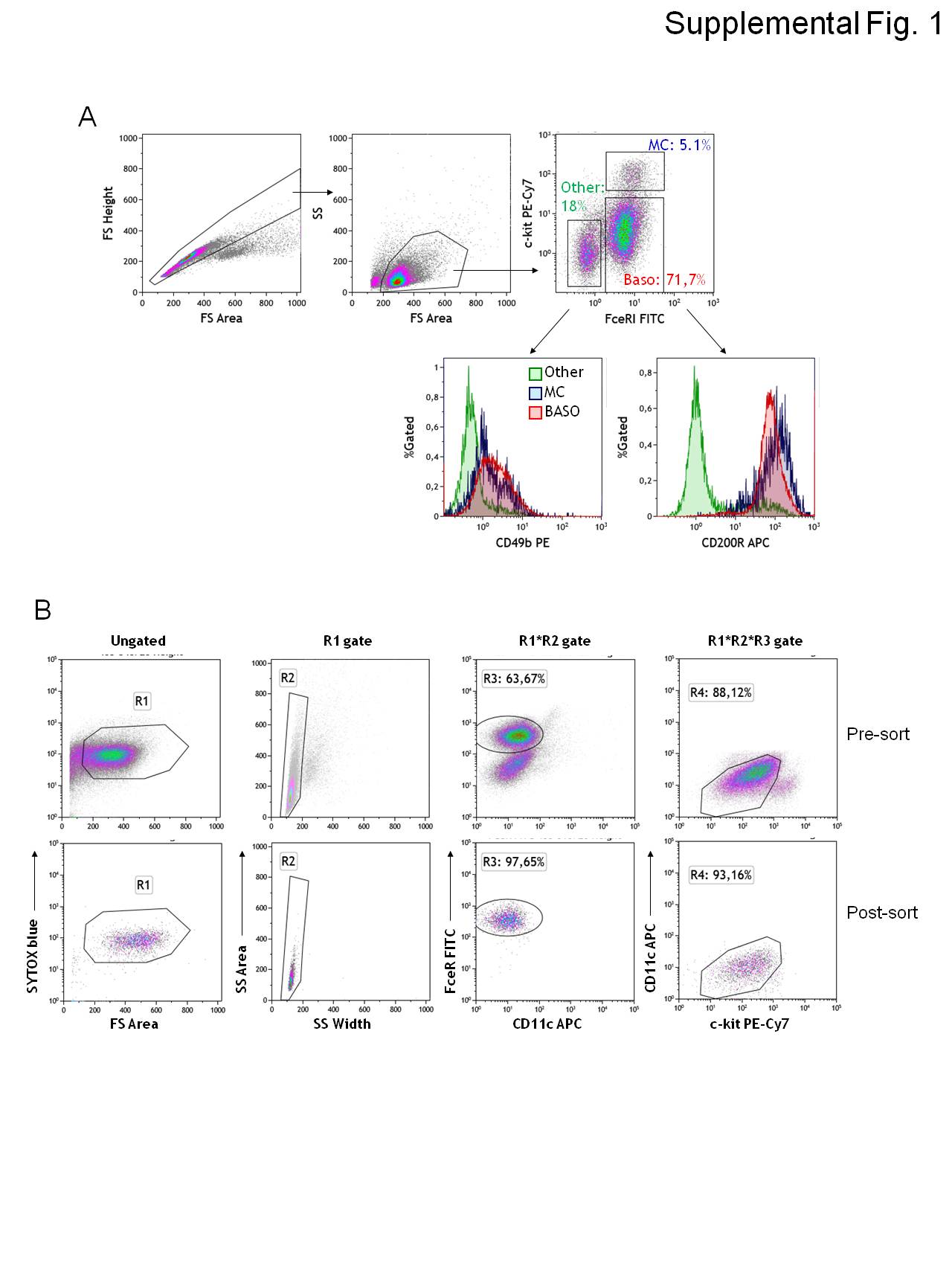

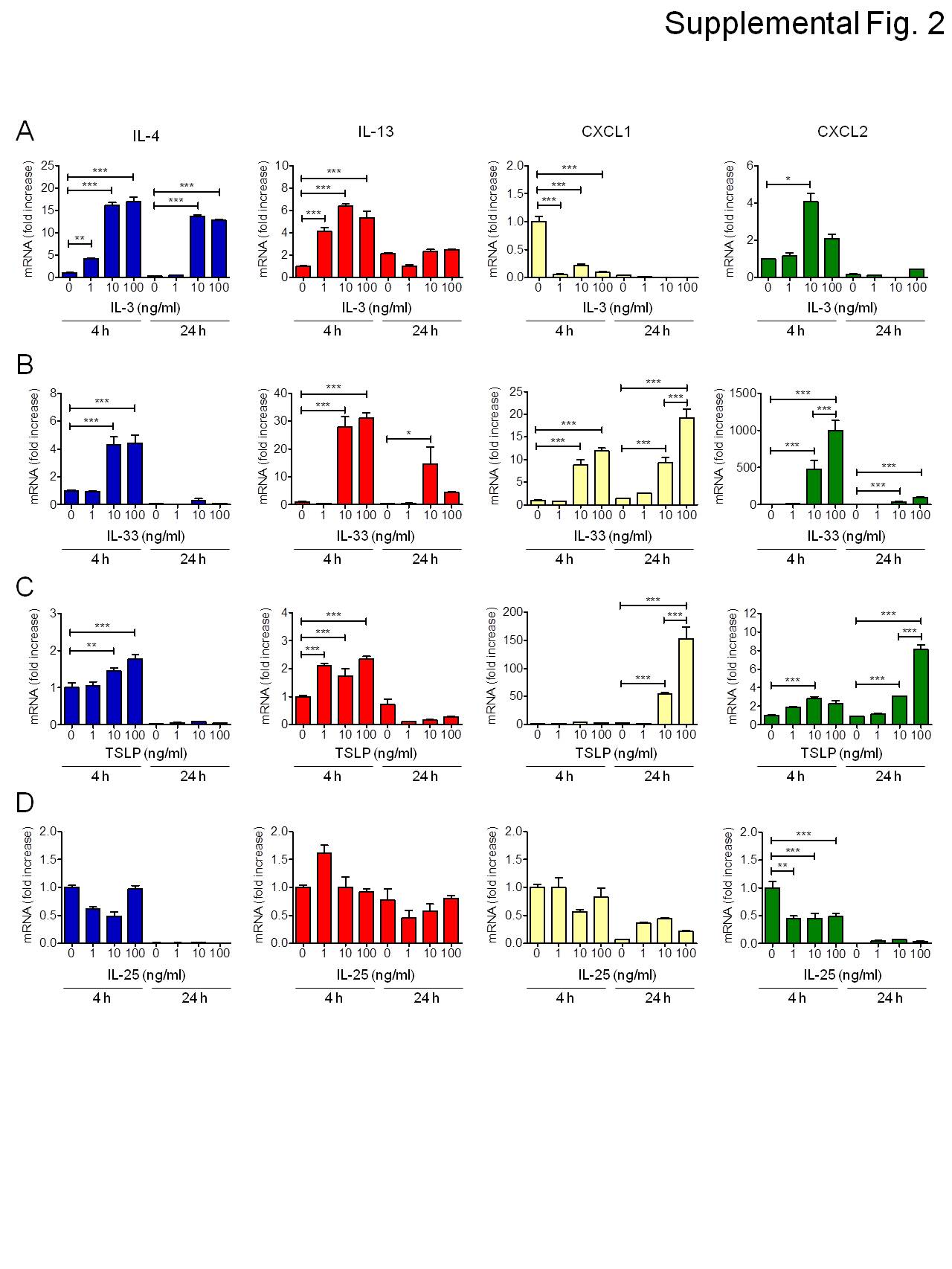

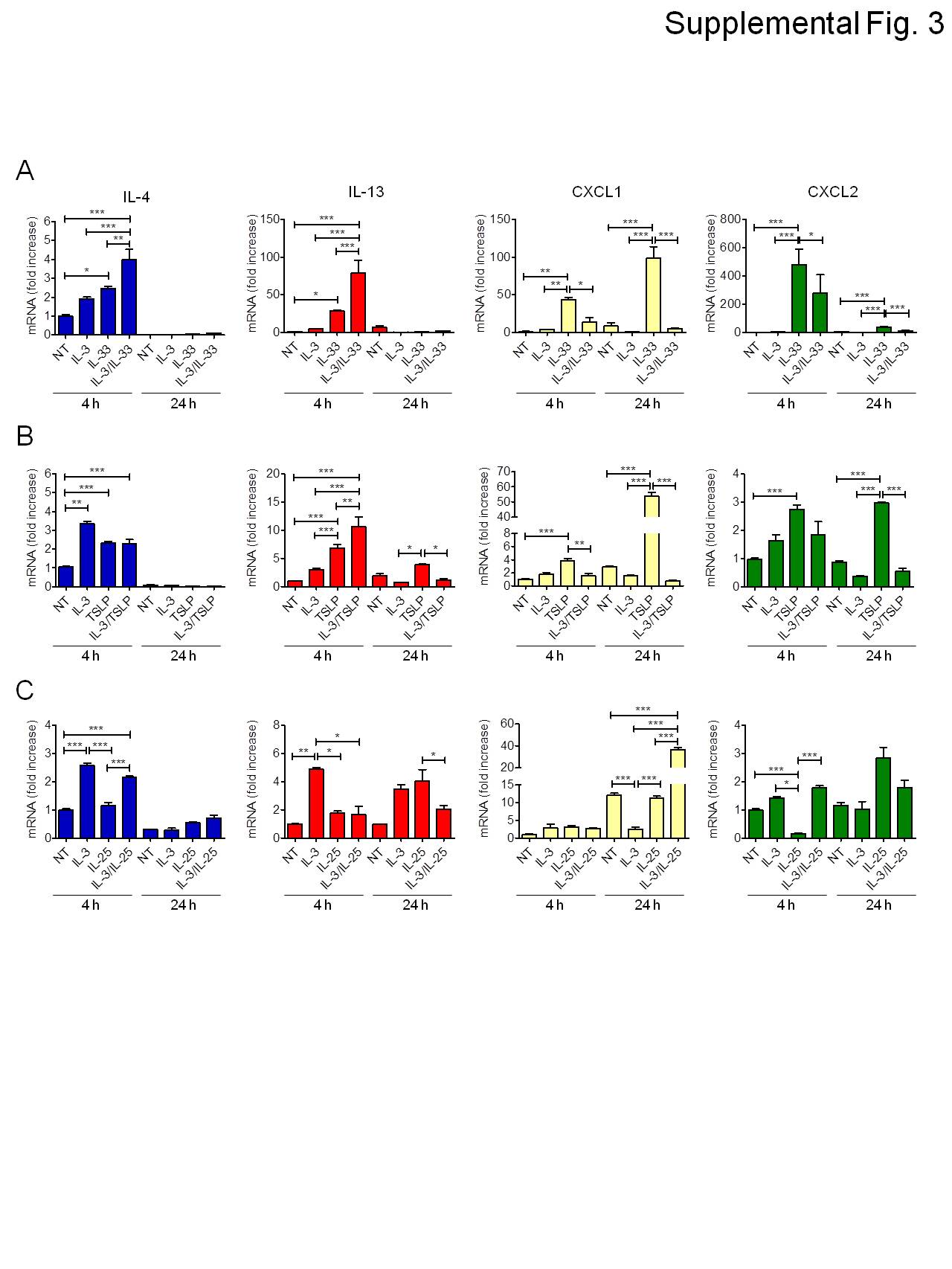


**Supplementary Figure Legends**

**Supplementary Figure 1.** (**A**) Phenotype of bone marrow-derived mBaso. Mouse bone marrow cells cultured in the presence of IL-3 (2 ng/ml) for 10 days yield around 70% basophils 5% mast cells and 18% of other bone marrow-derived cells. Both mBaso and mast cells express FcεRI, CD49b and CD200R3, while they can be distinguished by c-kit expression: mast cells are c-kit^+^, whereas mBaso are c-kit^-^. (**B**) Sorting strategy and purity of mBaso based on FcεR positivity, CD11c negativity and low expression of c-kit. SYTOX blue staining was applied to exclude dead cells.

**Supplementary Figure 2.** Effects of rmIL-3, rmIL-33, rmTSLP, or rmIL-25 on cytokine (IL-4, IL-13) and chemokine (CXCL1, CXCL2) mRNA expression in mBaso. Purified mBaso were stimulated with the indicated concentrations of rmIL-3 (**A**), rmIL-33 (**B**), rmTSLP (**C**), or rmIL-25 (**D**). Expression of IL-4, IL-13 and CXCL1 and CXCL2 mRNAs after 4 and 24 hours of stimulation was assessed by qRT-PCR. Data represent the mean fold increase of mRNA normalized to HPRT and with respect to untreated (NT) controls at 4 h +/- SD. One representative experiment out of at least three replicates is shown. **p* < 0.05; ***p* < 0.01; ****p* < 0.001.

**Supplementary Figure 3.** Effects of rmIL-3 alone and in combination with rmIL-33, rmTSLP, or rmIL-25 on cytokine (IL-4, IL-13) and chemokine (CXCL1, CXCL2) mRNA expression in mBaso. Purified mBaso were stimulated with rmIL-3 (1 ng/ml) alone and in combination with (**A**) rmIL-33 (10 ng/ml), (**B**) rmTSLP (10 ng/ml) or (**C**) rmIL-25 (10 ng/ml). mRNA expression was assessed by qRT-PCR after 4 and 24 hours of stimulation. Data represent the mean fold increase of mRNA normalized to HPRT and with respect to untreated (NT) controls at 4 h +/- SD. One representative experiment out of at least two replicates is shown. **p* < 0.05; ***p* < 0.01; ****p* < 0.001.


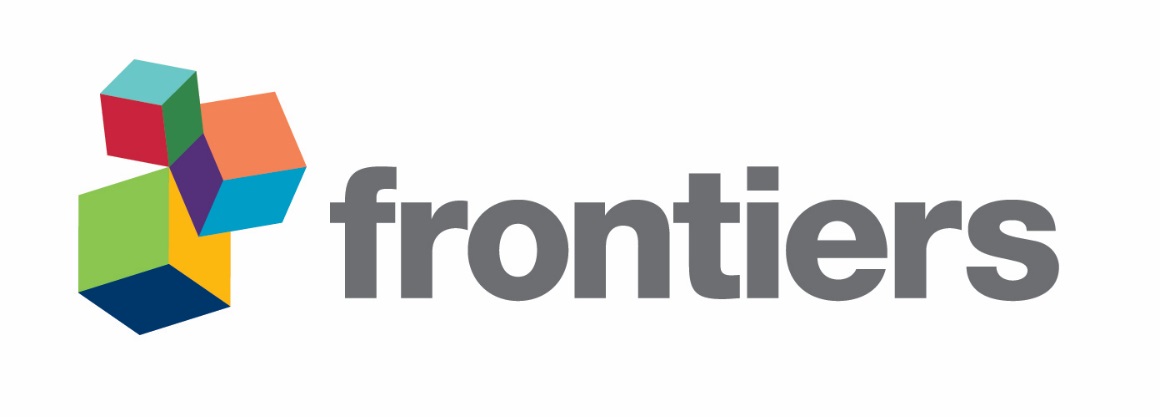

Supplement: Supplementary file 1 [file DataSheet_1.docx]
